# Lithium diffusion in Li_5_FeO_4_

**DOI:** 10.1038/s41598-018-24168-7

**Published:** 2018-04-11

**Authors:** Navaratnarajah Kuganathan, Poobalasuntharam Iyngaran, Alexander Chroneos

**Affiliations:** 1 Department of Materials, Imperial College London, London, SW7 2AZ United Kingdom; 2 Depratment of Chemistry, University of Jaffna, Sir Pon Ramanathan Road, Thirunelvely, Jaffna, Sri Lanka; 3 Faculty of Engineering, Environment and Computing, Coventry University, Priory Street, Coventry, CV1 5FB United Kingdom

## Abstract

The anti-fluorite type Li_5_FeO_4_ has attracted significant interest as a potential cathode material for Li ion batteries due to its high Li content and electrochemical performance. Atomic scale simulation techniques have been employed to study the defects and Li ion migration in Li_5_FeO_4_. The calculations suggest that the most favorable intrinsic defect type is calculated to be the cation anti-site defect, in which Li^+^ and Fe^3+^ ions exchange positions. Li Frenkel is also found to be lower in this material (0.85 eV/defect). Long range lithium diffusion paths were constructed in Li_5_FeO_4_ and it is confirmed that the lower migration paths are three dimensional with the lowest activation energy of migration at 0.45 eV. Here we show that doping by Si on the Fe site is energetically favourable and an efficient way to introduce a high concentration of lithium vacancies. The introduction of Si increases the migration energy barrier of Li in the vicinity of the dopant to 0.59 eV. Nevertheless, the introduction of Si is positive for the diffusivity as the migration energy barrier increase is lower less than that of the lithium Frenkel process, therefore the activation energy of Li diffusion.

## Introduction

The ever increasing requirements for better capacity, safety, cycle performance, and durability led to solid-state lithium batteries with the research focusing mainly on the electrolyte and cathode materials^1–12^. In that respect, considerable effort has been devoted to identify alternative cathode materials for rechargeable lithium ion batteries in order to provide high energy density for large scale applications particularly in electric vehicles and to replace conventional positive electrode material LiCoO_2_ due to its issues associated with cost and safety^13^. Promising positive electrode materials require meeting various essential conditions such as safety requirements, a relatively low cost, and large density of Li^+^ ions leading to a higher energy density.

Polyanion based olivine-structured orthophosphate LiFePO_4_ has attracted considerable attention as viable alternative to LiCoO_2_ due to its high electrochemical stability and iron, phosphorous are relatively safe, abundant and low-cost^14,15^. Though LiFePO_4_ is now in commercial use, several other promising cathode materials identified in recent years include Li_2_FeSiO_4_^16–18^, Li_2_MnSiO_4_^19,20^, LiFeBO_3_^21^, LiFeSO_4_F^22^, Li_2_Fe(SO_4_)_2_^23^, Li_2_FePO_4_F^24^, Li_2_FeP_2_O_7_^25^, Li_2_MnO_3_^26^, and Li_7_Mn(BO_3_)_3_^27^. Among these, “Li rich” Li_7_Mn(BO_3_)_3_ displayed an extremely large theoretical capacity (≈288 mAhg^−1^) upon extraction of three lithium ions per formula unit. Another “Li-rich” antifluorite Li_5_FeO_4_ has been reported as a promising cathode material for lithium ion batteries as it provides a high concentration of Li^+^ ions with a theoretical capacity of 867 mAhg^−1 ^^28–32^. Four Li^+^ ions have been extracted per formula unit, electrochemically between 3.5 and 4.5 V, with the evidence of partial oxidation of Fe^3+^ to Fe^4+^ in the X-ray absorption spectroscopy during the initial charge^30^. There is no evidence of Fe^3+^ to Fe^4+^ oxidation in the delithiated samples but a change in coordination of the Fe^3+^ ion from tetrahedral to octahedral coordination has been observed. This suggested that lithium extraction is predominantly assisted by the release of oxygen with the net loss of Li_2_O leaving Fe_2_O_3_ rich residual product^30^.

Atomic scale modeling techniques are powerful tools to provide detailed information about the defect chemistry and Li ion migration pathways together with the activation barrier providing complementary information to experiment. In the literature, no attempts have been made to study the defect process and Li ion diffusion in Li_5_FeO_4_ theoretically. The present study uses well-established atomistic modeling techniques to carry out a detailed survey of the relative energetics of the formation of intrinsic defects and the possible pathways for lithium ion conduction in Li_5_FeO_4_.

## Results and Discussion

### Structure and intrinsic defects

**Figure 1:**
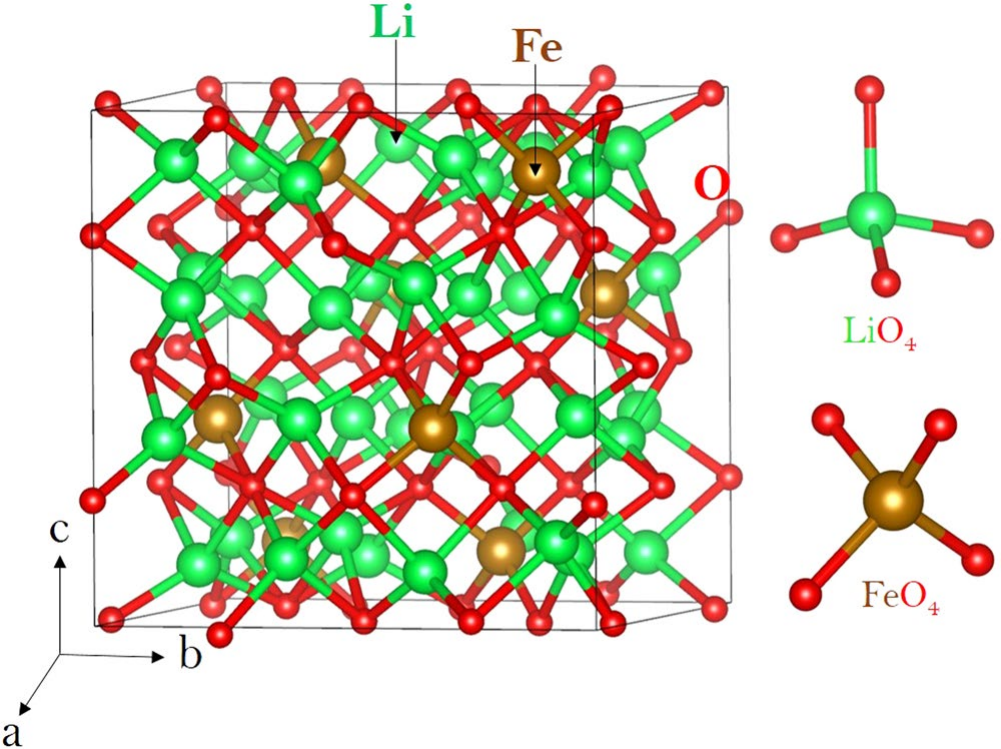
Crystal structure of Li_5_FeO_4_ (space group *Pbca*).

Crystal structure of Li_5_FeO_4_ exhibits a defect antifluorite structure with orthorhombic symmetry (space group *Pbca*). Experimentally determined lattice parameters are: a = 9.218, b = 9.213 and c = 9.153 Å^33^. Figure 1 shows the structure and the chemical environments of Li (forming a tetrahedron with four O atoms) and iron (forming a tetrahedron with four O atoms). Energy minimization calculations were performed on antifluorite bulk structure of Li_5_FeO_4_ to obtain the equilibrium lattice constants, thereby enabling an assessment (through comparison with experiment) of the quality of the pair potentials used in this study. The calculated equilibrium lattice constants (tabulated in Table S1) are in good agreement with experiment within a margin of 1% error.

A series of isolated point defect (vacancy and interstitial) energies were calculated, which were combined to determine the formation energies for Frenkel and Schottky-type defects in Li_5_FeO_4_. The equations represent the reactions involving these defects as written using Kröger-Vink notation and corresponding reaction energies are tabulated in Table 1. The most favorable intrinsic disorder is found to be the Li-Fe anti-site defect (equation 7 of Table 1). The exact concentration is dependent on the temperature and synthetic routes. The formation of Li Frenkel is the second most favorable process in this material. Fe Frenkel, O Frenkel and Schottky defects are highly unfavorable and thus unlikely to occur in any significant concentration in Li_5_FeO_4_.

**Table 1:** Energetics of intrinsic defects in Li_5_FeO_4_.

Defect Process	Equation	Defect energy (eV)	Defect energy (eV)/defect
Li Frenkel/1	$L{i}_{Li}^{X}\to \,{V^{\prime} }_{Li}+L{i}_{i}^{\bullet }$	1.70	0.85
O Frenkel/2	${O}_{O}^{X}\to {V}_{O}^{\bullet \bullet }+\,{O^{\prime\prime}}_{i}$	7.23	3.62
Fe Frenkel3	$F{e}_{Fe}^{X}\to {V^{\prime\prime\prime}}_{Fe}+F{e}_{i}^{\bullet \bullet \bullet }$	8.66	4.33
Schottky/4	$$\begin{array}{c}5\,L{i}_{Li}^{X}+\,F{e}_{Fe\,}^{X}+4{O}_{O}^{X\,}\to 5{V^{\prime} }_{Li}+{V^{\prime\prime\prime}}_{Fe}+\\ 4{V}_{O}^{\bullet \bullet }+L{i}_{5}Fe{O}_{4}\end{array}$$	25.40	2.54
Li_2_O Schottky- like/5	$\,2\,L{i}_{Li}^{X}+{O}_{O}^{X\,}\to 2{V^{\prime}}_{Li}+{V}_{O}^{\bullet \bullet }+L{i}_{2}O$	5.83	1.94
Fe_2_O_3_ Schottky- like/6	$2F{e}_{Fe\,}^{X}+3{O}_{O}^{X\,}\to \,2\,{V^{\prime\prime\prime}}_{Fe}\,+3{V}_{O}^{\bullet \bullet }+F{e}_{2}{O}_{3}$	20.72	4.14
Li/Fe anti-site (isolated)/7	$L{i}_{Li}^{X}+F{e}_{Fe}^{X\,}\to {Li^{\prime\prime}}_{Fe}+F{e}_{Li}^{\bullet \bullet }$	4.26	2.13
Li/Fe anti-site (cluster)/8	$L{i}_{Li}^{X}+F{e}_{Fe}^{X\,}\to \{{Li^{\prime\prime}}_{Fe}:F{e}_{Li}^{\bullet \bullet }\}X$	1.12	0.56

### Lithium ion diffusion

The lithium ion mobility in the Li_5_FeO_4_ material is of crucial importance when assessing its use as a possible high-rate cathode material in lithium batteries. Also it is important to observe the Li ion migration energies with paths in this material. However, the diffusion paths in the Li_5_FeO_4_ structures have not been established experimentally.

**Figure 2:**
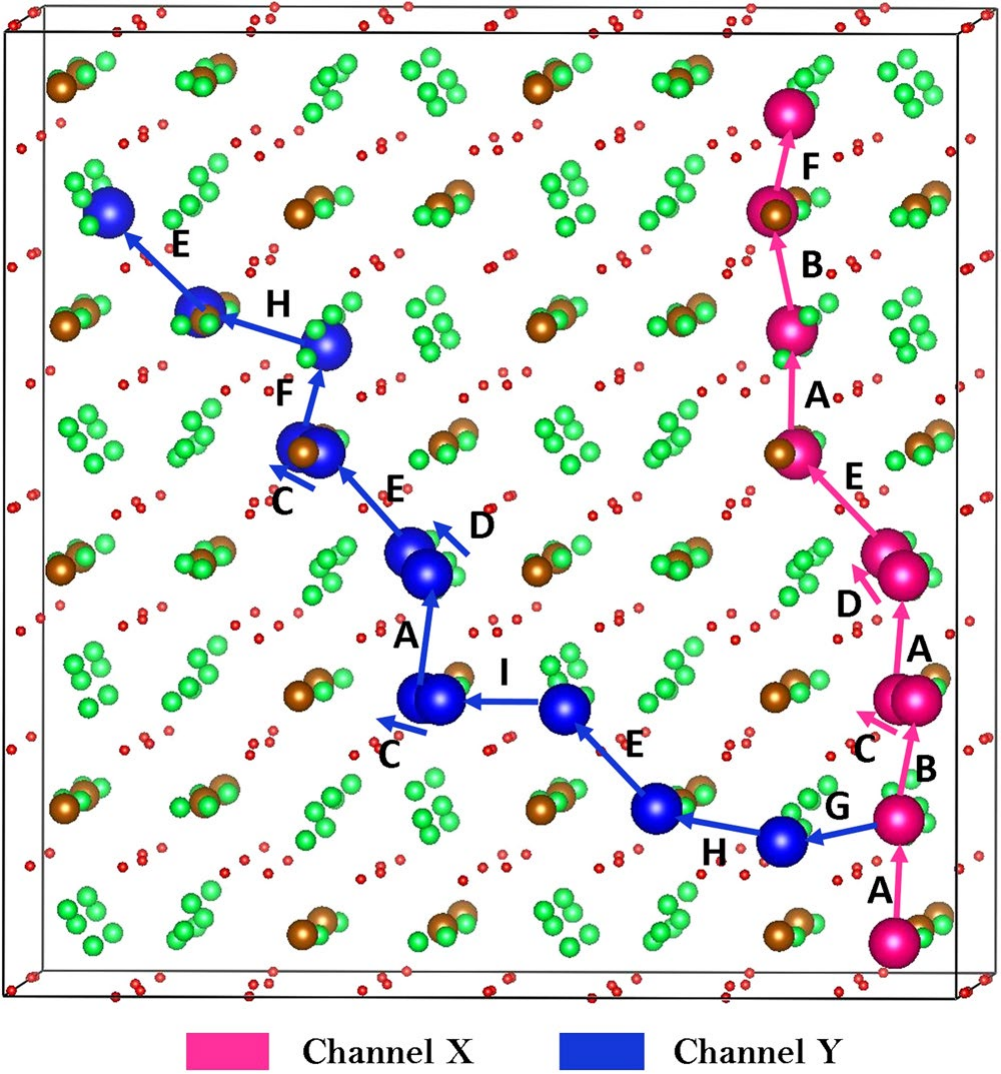
Possible long range lithium vacancy migration paths considered. Green, brown and red colors correspond to Li, Fe and O atoms respectively. Larger spheres (blue and pink) correspond to Li ions and were used to construct different three dimensional channels.

**Figure 3:**
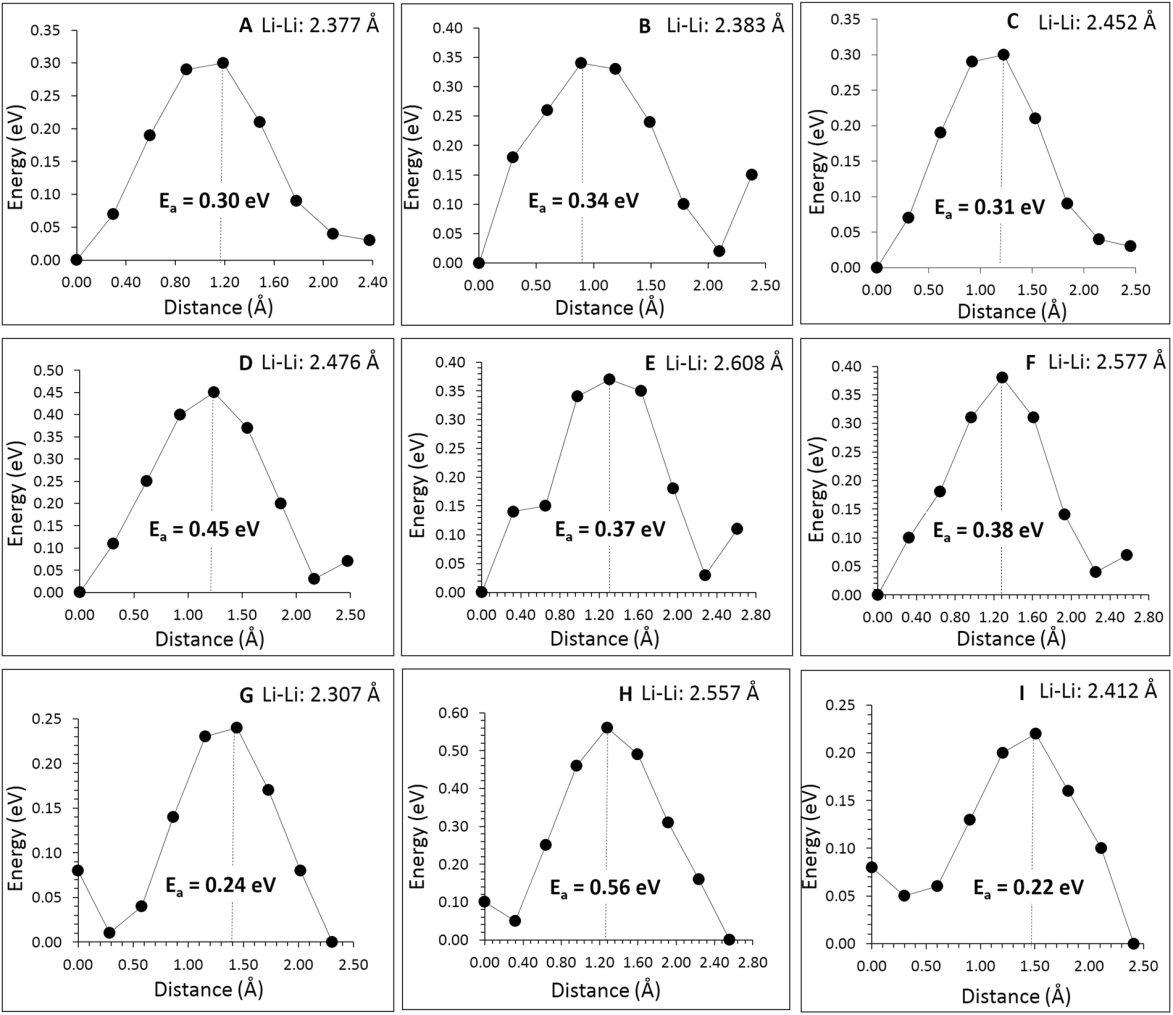
Nine different energy profiles [as shown in Fig. 2] of Li vacancy hopping between two adjacent Li sites in Li_5_FeO_4_.

Atomistic simulation techniques enable the examination of various possible diffusion paths responsible for lithium ion conduction. We have identified two main long range diffusion channels connecting local Li hops, illustrated as X and Y in Fig. 2. In both channels Li ions can also diffuse in opposite directions as well. The lowest overall activation energy is calculated at 0.45 eV for the X channel. The second lowest activation energy channel, Y, has an overall activation barrier of 0.56 eV. Individual Li-Li separations and corresponding activation energy barriers are tabulated in Table 2. We have considered a range of other paths with longer Li-Li migration distances of >2.70 Å, but these revealed high migration barriers (>0.85 eV). Potential energy profile diagrams showing the activation energies are shown in Fig. 3.

**Table 2:** Calculated Li-Li separations and activation energies for the lithium ion migration between two adjacent Li sites refer to Fig. 2.

Migration path	Li-Li separation (Å)	Activation energy (E_a_)(eV)
A	2.377	0.30
B	2.383	0.34
C	2.452	0.31
D	2.476	0.45
E	2.608	0.37
F	2.577	0.38
G	2.307	0.24
H	2.557	0.56
I	2.412	0.22

### Tetravalent doping

There is a low migration activation energy for the migration of lithium via the vacancy mechanism (0.45 eV), however, the Li Frenkel energy (0.85 eV/defect) will limit the concentration of *V*′_*Li*_. The latter are important as they effectively act as the vehicles mediating Li self-diffusion. A way to increase the concentration of *V*′_*Li*_ is by the introduction of tetravalent dopant atoms via the solution of other oxides in Li_5_FeO_4_. This processes can be described as (in the Kröger-Vink notation):

1 $$2R{O}_{2}+2F{e}_{Fe}^{X}+2L{i}_{Li}^{X}\to 2{R}_{Fe}^{\bullet }+2{V}_{Li}^{\text{'}}+F{e}_{2}{O}_{3}+L{i}_{2}O.$$

**Figure 4:**
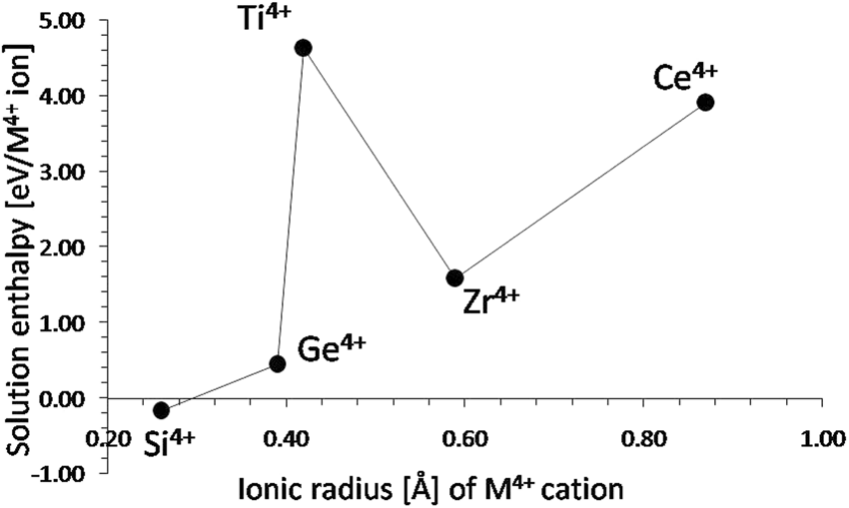
Enthalpy of solution of RO_2_ (*R* = Ce, Zr, Ti, Si and Ge) with respect to of the R^4+^ ionic radius in Li_5_FeO_4_.

Analogous defect engineering strategies have been introduced in order to enhance the concentration of vacancy defects in oxides^34^. We considered the solution *RO*_2_ oxides (*R = *Ce, Zr, Ti, Si and Ge), aiming to find an oxide with a low solution enthalpy. As it can be observed in Fig. 4 the solution energy of SiO_2_ is the lowest one (−0.16 eV/defect) and interestingly it is negative. This in turn implies that the solution of SiO_2_ in Li_5_FeO_4_ is energetically favourable leading to the formation of a non-equilibrium concentration of *V*′_*Li*_. Additionally, the solution of GeO_2_, although positive (0.46 eV/defect), is lower in energy and thus more energetically favourable as compared to the Li Frenkel. In essence doping with Si or Ge will result in the formation of *V*′_*Li*_without the higher energies required by the Li Frenkel reaction. Importantly, these vacancies will be vehicles for Li self-diffusion, increasing the Li diffusivity. Given that the solution enthalpies for SiO_2_ and GeO_2_ will be lower than the enthalpies for Schottky and Frenkel disorder, the *V*′_*Li*_will be prevalent due to this extrinsic processes.

**Figure 5:**
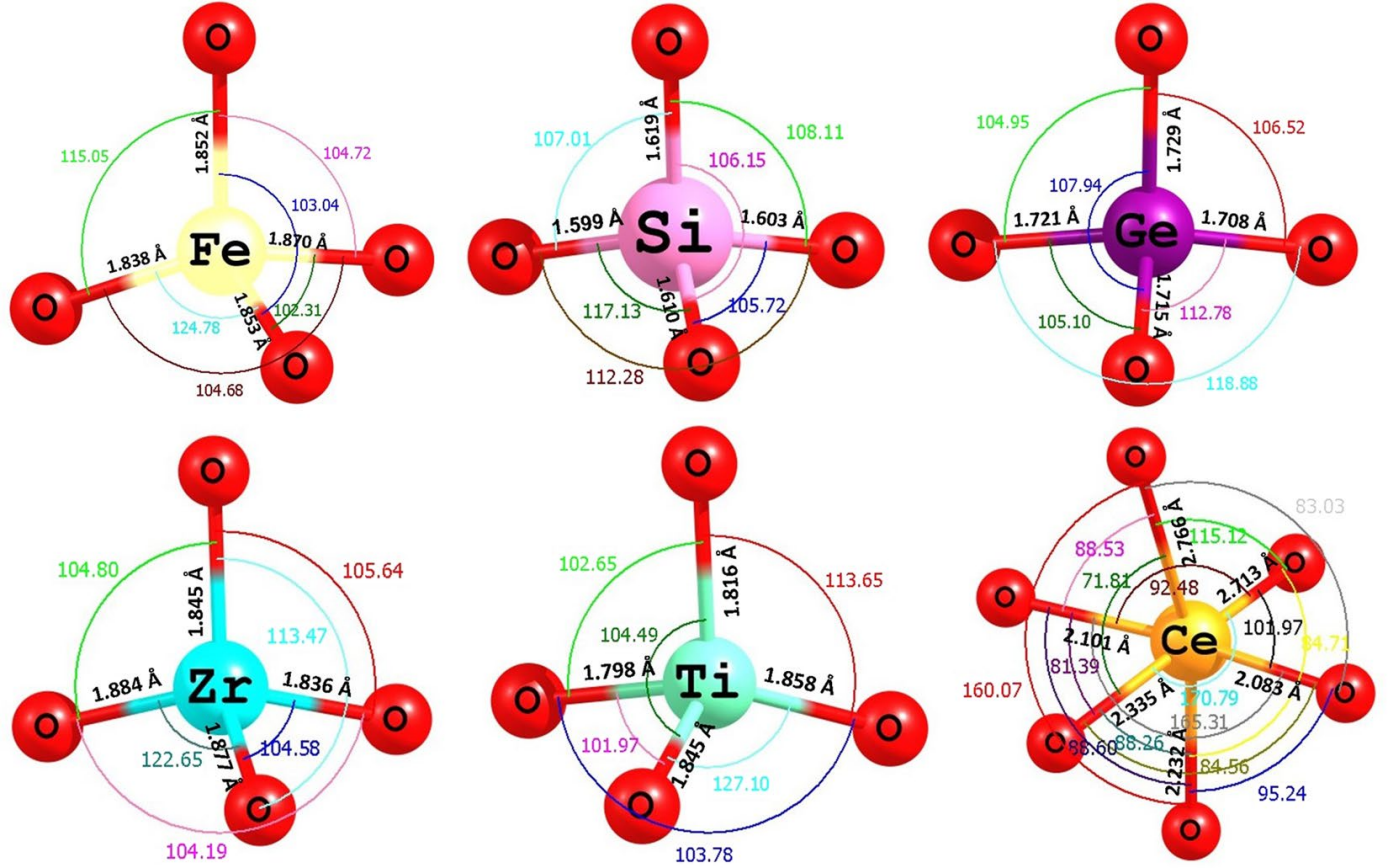
Tetrahedral FeO_4_ unit in the relaxed structure of undoped Li_5_FeO_4_ and the coordination formed by the dopants on the Fe site with neighbor oxygen.

In the Li_5_FeO_4_ crystal structure Fe forms a tetrahedral coordination. Fig. 5 shows the local coordination of dopants together with the bond lengths and bond angles in the relaxed structures. For comparison, bond lengths and bond angles of the FeO_4_ unit in the relaxed structure of Li_5_FeO_4_ are also given. In most silicates, a SiO_4_ structure is formed with four O atoms in tetrahedral coordination around the Si atom. A similar feature is observed in some solids containing Ge. This reflects in the solution energies. Lower solution energies were calculated for Si and Ge. Ti and Zr also form tetrahedral coordination but their solution energies are high. This can be due to their unusual tetrahedral coordination as Zr and Ti normally form octahedral six-coordinate complexes in their crystal structures. Ce clearly forms a distorted octahedral coordination revealing a high positive solution enthalpy.

**Figure 6:**
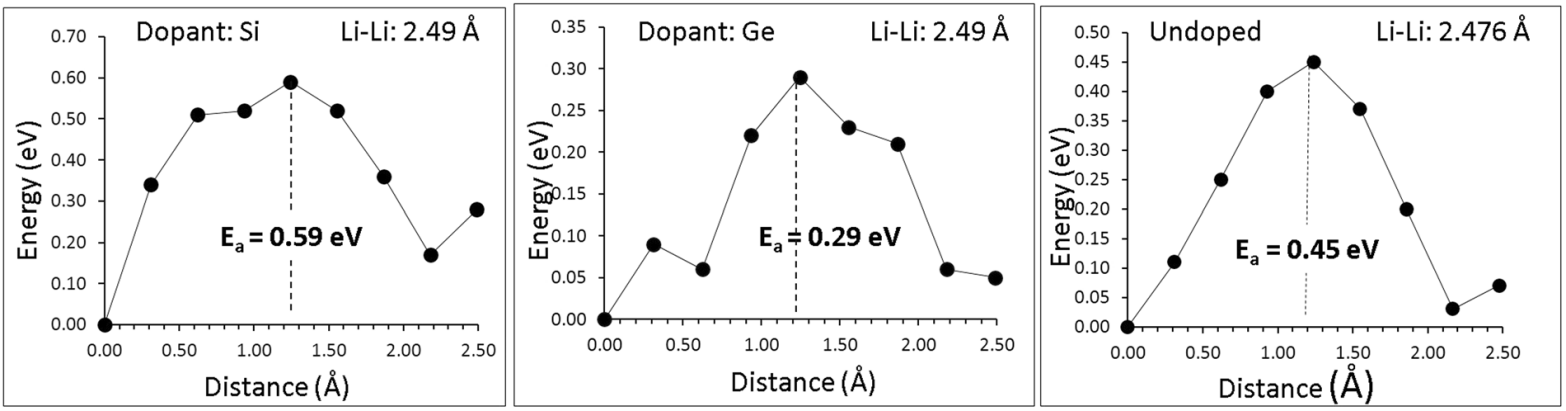
Energy profile diagrams for the Li vacancy hoping closer to the dopants (Si and Ge) on the Fe site and in the absence of dopants (undoped).

Introducing Si or Ge dopants in Li_5_FeO_4_ will have an effect on the migration energies of lithium. Fig. 6 represents the impact of Si or Ge dopants on the migration barrier of Li. The presence of the Si substitutional will increase the migration energy barrier of Li by 0.14 eV (i.e. to 0.59 eV). Interestingly, the Ge substitutional will lead to the reduction of the migration energy barrier to 0.29 eV. Considering the activation energy of the diffusion process (formation energy + migration energy) it can be concluded that in undoped Li_5_FeO_4_ the activation energy is 1.30 eV, in GeO_2_ doped Li_5_FeO_4_ it is 0.75 eV and finally, in SiO_2_ doped Li_5_FeO_4_ only 0.59 eV (as the solution energy is negative we assume that there will be an non-equilibrium concentration of *V*′_*Li*_ so their formation energy will be 0).

### Summary

Atomistic simulation techniques have been employed to provide detailed insights into intrinsic defects and lithium ion mobility in Li_5_FeO_4_. Our simulations reproduce the experimentally observed crystal structure of Li_5_FeO_4_. The most favorable intrinsic disorder type is the Li-Fe anti-site defect. This suggests that there will be a population of Li ion on Fe sites and Fe on Li sites. Frenkel and Schottky type disorders (except Li Frenkel) are highly unfavorable. The lowest activation energy for Li migration in Li_5_FeO_4_ is 0.45 eV. Considering the formation energy of vacancies via the Li Frenkel energy the activation energy of the process becomes 1.3 eV. The solution of SiO_2_ effectively forms *V*′_*Li*_ but can increase the migration energy barrier to 0.59 eV. There is therefore an activation energy reduction as compared to the undoped case of 0.71 eV. Finally, through the solution of GeO_2_ only 0.46 are required for the formation of every *V*′_*Li*_, whereas there is a reduced migration energy barrier (0.29 eV) for Li in the vicinity of the Ge substitutionals. The activation energy of diffusion is 0.75 eV that is 0.55 eV lower as compared to the undoped case and only 0.16 eV higher as compared to Si-doped Li_5_FeO_4_. We propose experimental investigations and diffusion studies in Li_5_FeO_4_ doped with Si and or Ge. A key objective of the present investigation is to motivate experimental and theoretical studies^35–37^ to determine the defect processes of anti-fluorite materials such as Li_5_FeO_4_ and their potential application as energy materials.

## Methods

In order to calculate the energetics for the formation of intrinsic defects and possible Li ion diffusion pathways, the classical pair potential method as implemented in the GULP package was employed^38^. This method is based on the classical Born model description of an ionic crystal lattice. All systems were treated as crystalline solids with interactions between ions consisting of the long-range attractions and short-range repulsive forces representing electron-electron repulsion and van der Waals interactions. The short range interactions were modelled using Buckingham potentials (refer to Table S2). Simulation boxes and the corresponding atom positions were relaxed using the Broyden-Fletcher-Goldfarb-Shanno (BFGS) algorithm^39^. The Mott-Littleton method^40^ was used to investigate the lattice relaxation about point defects and the migrating ions. It divides the crystal lattice into two concentric spherical regions, where the ions within the inner spherical region (on the order of >700 ions) immediately surrounding the defect relaxed explicitly. All defect calculations were performed using a perfect 2 × 2 × 2 supercell containing 640 atoms. Li ion diffusion was calculated considering two adjacent vacancy sites as initial and final configurations. Seven interstitial Li ions were considered in a direct linear route and they were fixed while all other ions were free to relax. The local maximum energy along this diffusion path is calculated and reported as activation energy. As the present model assumes a full charge ionic model with the calculations corresponding to the dilute limit the defect enthalpies will be overestimated, however, relative energies and trends will be consistent.

## Electronic supplementary material

Supplementary Information
