# Supplementary material for: Lithium diffusion in Li5FeO4
Source: Sci Rep. 2018 Apr 11;8:5832. doi: 10.1038/s41598-018-24168-7 (PMC5895795; doi:10.1038/s41598-018-24168-7)
Supplement: Supplementary file 1 — Supplementary Information [file 41598_2018_24168_MOESM1_ESM.docx]

**Lithium diffusion in Li_5_FeO_4_**

Navaratnarajah Kuganathan^1^, Poobalasuntharam Iyngaran^2^, Alexander Chroneos^1,3^

^1^Department of Materials, Imperial College London, London, SW7 2AZ, United Kingdom

^2^Depratment of Chemistry, University of Jaffna, Sir Pon Ramanathan Road, Thirunelvely, Jaffna, Srilanka

^3^Faculty of Engineering, Environment and Computing, Coventry University, Priory Street, Coventry CV1 5FB, United Kingdom

**Table S1.** Calculated and experimental structural parameters for orthorhombic (*Pbca*) Li_5_FeO_4_

| Parameter | Calculated | Experiment | \|∆\|(%) |
| --- | --- | --- | --- |
| a (Å) | 9.1649 | 9.2180 | 0.58 |
| b (Å) | 9.1179 | 9.2130 | 1.03 |
| c (Å) | 9.1590 | 9.1530 | 0.06 |
| α = β = γ (°) | 90.00 | 90.00 | 0.00 |
| Volume (Å^3^) | 775.507 | 777.832 | 0.30 |

**Table S2**. Interatomic potential parameters used in the atomistic simulations of Li_5_FeO_4_

Two-body [Φ*_ij_* (*r_ij_*) = *A_ij_* exp (−*r_ij_*/*ρ_ij_*) −*C_ij_ / r_ij_*^6^]

| Interaction | *A* (eV) | *ρ*(Å) | *C* (eV·Å^6^) | *Y* (e) | K (eV·Å^-2^) |
| --- | --- | --- | --- | --- | --- |
| Li^+^ - O^2−^ | 632.1018 | 0.2906 | 0.000 | 1.000 | 99999 |
| Fe^3+^ - O^2−^ | 1156.36 | 0.3299 | 0.000 | 4.970 | 304.7 |
| O^2−^ - O^2−^ | 22764.30 | 0.1490 | 43.00 | −2.240 | 42.00 |
| Si^4+^ - O^2−^ | 1283.91 | 0.32052 | 10.66 | 4.00 | 99999 |
| Ge^4+^ - O^2−^ | 1497.3996 | 0.325646 | 16.000 | 4.00 | 99999 |
| Zr^4+^ - O^2−^ | 985.869 | 0.3760 | 0.000 | 1.35 | 169.617 |
| Ce^4+^ - O^2−^ | 1986.83 | 0.3511 | 20.40 | 7.70 | 291.75 |
| Ti^4+^ - O^2−^ | 5111.7 | 0.2625 | 0.000 | -0.10 | 314.0 |


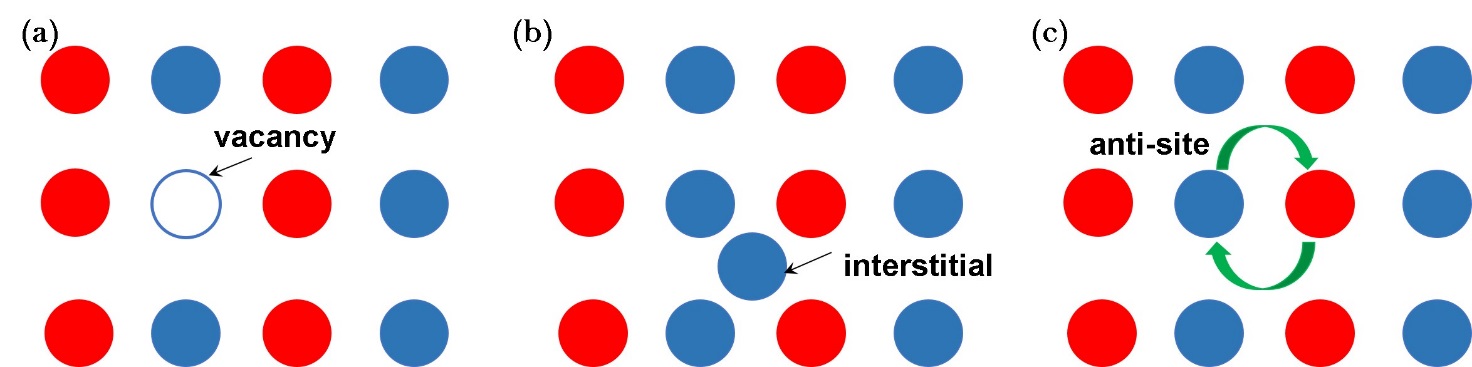


**Figure S1**. Models showing (a) vacancy (b) interstitial and (c) anti-site defects in a crystal structure
